# Phase Ia study of the indoleamine 2,3-dioxygenase 1 (IDO1) inhibitor navoximod (GDC-0919) in patients with recurrent advanced solid tumors

**DOI:** 10.1186/s40425-018-0351-9

**Published:** 2018-06-20

**Authors:** Asha Nayak-Kapoor, Zhonglin Hao, Ramses Sadek, Robin Dobbins, Lisa Marshall, Nicholas N. Vahanian, W. Jay Ramsey, Eugene Kennedy, Mario R. Mautino, Charles J. Link, Ray S. Lin, Stephanie Royer-Joo, Xiaorong Liang, Laurent Salphati, Kari M. Morrissey, Sami Mahrus, Bruce McCall, Andrea Pirzkall, David H. Munn, John E. Janik, Samir N. Khleif

**Affiliations:** 1 Georgia Cancer Center at Augusta University, Augusta, GA USA; 2 NewLink Genetics, Ames, IA USA; 3 Genentech, Inc., South San Francisco, CA USA; 4 Present Address: Lombardi Comprehensive Cancer Center, Georgetown University School of Medicine, 3900 Reservoir Rd NW, Washington, DC, 20007 USA

**Keywords:** Phase I, IDO1, Navoximod, Kynurenine, Tryptophan

## Abstract

**Background:**

Indoleamine-2,3-dioxygenase 1 (IDO1) catalyzes the oxidation of tryptophan into kynurenine and is partially responsible for acquired immune tolerance associated with cancer. The IDO1 small molecule inhibitor navoximod (GDC-0919, NLG-919) is active as a combination therapy in multiple tumor models.

**Methods:**

This open-label Phase Ia study assessed safety, pharmacokinetics (PK), pharmacodynamics (PD), and preliminary anti-tumor activity of navoximod in patients with recurrent/advanced solid tumors, administered as 50-800 mg BID on a 21/28 day and at 600 mg on a 28/28 day schedule. Plasma kynurenine and tryptophan were longitudinally evaluated and tumor assessments were performed.

**Results:**

Patients (*n* = 22) received a median of 3 cycles of navoximod. No maximum tolerated dose was reached. One dose-limiting toxicity of Grade 4 lower gastrointestinal hemorrhage was reported. Adverse events (AEs) regardless of causality in ≥20% of patients included fatigue (59%), cough, decreased appetite, and pruritus (41% each), nausea (36%), and vomiting (27%). Grade ≥ 3 AEs occurred in 14/22 patients (64%), and were related to navoximod in two patients (9%). Navoximod was rapidly absorbed (T_max_ ~ 1 h) and exhibited dose-proportional increases in exposure, with a half-life (t_1/2_ ~ 11 h) supportive of BID dosing. Navoximod transiently decreased plasma kynurenine from baseline levels with kinetics consistent with its half-life. Of efficacy-evaluable patients, 8 (36%) had stable disease and 10 (46%) had progressive disease.

**Conclusions:**

Navoximod was well-tolerated at doses up to 800 mg BID decreasing plasma kynurenine levels consistent with its half-life. Stable disease responses were observed.

**Trial registration:**

ClinicalTrials.gov identifier: NCT02048709.

## Background

Indoleamine 2,3-dioxygenase 1 (IDO1) is a cytosolic enzyme that catalyzes the first and rate-limiting step in the oxidation of L-tryptophan (Trp) into kynurenine (Kyn) [1]. IDO1 is constitutively expressed in many tissues where it regulates local inflammation and moderates response to foreign or uncommon non-pathological antigens. The IDO1 pathway contributes to local control of inflammation and participates in acquired immune peripheral tolerance to limit inflammation and prevent normal tissue injury.

In cancer cells, IDO1 mediates an acquired immunosuppression leading to local and systemic immune tolerance towards the tumor by helping to evade immune surveillance [2]. IDO1 has at least two methods for inducing immune suppression in T cells. First, depletion of Trp in the local tumor microenvironment activates a starvation response in T cells that impairs their function. Second, increase in levels of Kyn, an endogenous ligand for the aryl hydrocarbon receptor, suppresses effector T cells and hyperactivates Tregs. Together, these effects lead to decreased inflammation and immune responsivity [3, 4]. Inhibition of IDO1 activity reverts these effects, while also modulating the function of dendritic cells and skewing antigen presentation away from a tolerogenic phenotype.

IDO1 is expressed by many tumors including malignant melanoma [5, 6], pancreatic cancer [7], ovarian cancer [8, 9], acute myelogenous leukemia [10, 11], colorectal cancer [12, 13], prostate cancer [14], and endometrial cancer [15, 16]. Increased tumor expression of IDO1 has been associated with significantly worse clinical outcomes [1]. Recently, the IDO inhibitor epacadostat (INCB024360) showed reductions in plasma Kyn levels in an ex vivo assay in patients with advanced solid malignancies [17]. Combination studies examining IDO1 inhibitors given with checkpoint blockade via programmed cell death protein 1 (PD-1) or programmed death-ligand 1 (PD-L1) inhibition appears more promising in light of encouraging response rates and durability of responses [18–21], which is now being evaluated in Phase III clinical trials.

Navoximod (GDC-0919; previously NLG919) is an investigational small molecule inhibitor of IDO1 that was developed to treat the immune tolerance associated with cancer. Navoximod shows a potency (EC_50_) of 70 nM in cellular activity assays and 90 nM in human T cell-proliferation MLR assays [22]. In preclinical models, combination treatment of navoximod with chemotherapy, radiotherapy, or vaccines leads to improved antitumor response [23]. Treatment with navoximod and vaccination in B16F10 tumor-bearing mice increased the T cell response, leading to improved anti-tumor efficacy [22]. In preclinical combination studies with anti-PD-L1 and anti-OX40, inhibition of IDO1 showed greater effect in activating intratumoral CD8^+^ T cells and inhibiting tumor growth than either treatment alone [22, 24–26].

These data support the investigation of navoximod as a cancer immunotherapeutic agent. The primary objectives of this study were to evaluate the safety and tolerability of single-agent navoximod and to determine the maximum tolerated dose (MTD) in patients with recurrent advanced solid tumors. We also sought to characterize the pharmacokinetic (PK) properties of navoximod after single and repeat doses, and identify the single-agent recommended Phase II dose and schedule. Other objectives included assessment of pharmacodynamic (PD) modulation of plasma Kyn and Trp by navoximod, and an evaluation of response rates and duration of responses.

## Methods

### Study design

This was a single-arm, single-center, non-randomized Phase I open-label, dose-ranging study of navoximod (supplied by NewLink Genetics and later Genentech, Inc.) in patients with recurrent, advanced solid tumors. All patients were enrolled at the Georgia Cancer Center at Augusta University, Augusta, GA.

**Fig. 1:**
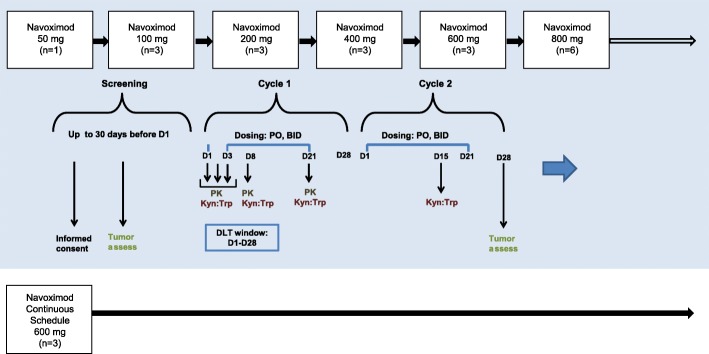
Study design for ascending (BID) and continuous schedules

Based on validated preclinical models to establish the starting dose of navoximod in line with standard Phase I oncology clinical trials, and predicted half-life, the navoximod starting dose was defined as 50 mg twice daily (BID), and after that dose was demonstrated to be safe, navoximod was escalated in a modified 3 + 3 design at doses of 100, 200, 400, 600, and 800 mg BID for 21 days, followed by 7 days without dose administration (21/28 day schedule). Navoximod was administered every 12 h on an empty stomach (no food or drink other than water for at least 2 h prior to dose and at least 1 h after dose). Having demonstrated good tolerability at the 21/28 schedule, a continuous dosing cohort of 600 mg BID for 28 days was added (28/28-day schedule). Figure 1 shows the study design, dose levels, timepoints for PK/PD, tumor assessments, and number of patients entered in each cohort. MTD was defined as the highest dose at which 2 out of 6 treated patients experienced Dose limiting Toxicity (DLT) in the first cycle. DLTs were defined as any clinically significant non-hematologic toxicity of Grade 3 or greater that occurred during the first cycle of 14 days (excluding Grade 3 nausea, vomiting, diarrhea managed with optimal medication) not related to underlying malignancy; an immune related adverse event (AE) that lasted more than 28 days (an AE associated with exposure to the study drug and that was consistent with an immune phenomenon); or hematological toxicity (Grade 4 neutropenia, Grade 3 or greater febrile neutropenia, or Grade 4 thrombocytopenia that persisted more than 5 days despite optimal management).

### Patients

Eligible patients were age ≥ 18 years with histologically confirmed solid tumors that were refractory to standard therapies or for which no approved or curative therapy was available, had Eastern Cooperative Oncology group (ECOG) performance status of 0-1, adequate hematologic and end organ function, and evaluable or measurable disease per RECIST v1.1. Patients were excluded if there was a history of autoimmune disease, prior treatment with any anti-cancer therapy, cytokines or toll-like receptors within 5 drug half-lives, antibodies directed at PD-1, PD-L1 or CTLA4, or other IDO inhibitors, prior malignancy other than non-melanoma skin cancer or carcinoma in situ of the cervix, infection with HIV, Hepatitis B or C, concomitant immunotherapy, untreated brain metastases, or current treatment with drugs associated with Torsades de Pointes, which could not be safely discontinued.

### Safety assessments

Safety assessments consisted of per-visit monitoring and recording AEs, including serious and non-serious AEs of special interest (cases of potential drug induced liver injury that included an elevated ALT or AST in combination with either an elevated bilirubin or clinical jaundice, as defined by Hy’s law, and any DLT). Protocol-specified laboratory assessments, were also performed. AEs were monitored and graded for severity using the National Cancer Institute Common Terminology Criteria for Adverse Events, version 4.0.

### Pharmacokinetic assessments

Frequent blood samples were taken for navoximod PK evaluations after single (Cycle 1, Day 1-3 [0-48 h]) and multiple twice-daily doses (Cycle 1, Day 21 [0-12 h]). Plasma concentrations of navoximod were determined using a validated liquid chromatographic-tandem mass spectrometry (LC/MS-MS) method with a lower limit of quantitation of 1 ng/mL. The LC/MS/MS method employed [^13^C, ^15^N_2_]-navoximod as the internal standard. Samples were processed using protein precipitation extraction procedures. Chromatography of navoximod was achieved with a Waters Atlantis T3 (50 × 4.6 mm, 5 μm) column. A Sciex API 4000 with an electrospray source in the positive ion multiple-reaction monitoring mode was used for detection of navoximod in human plasma. The dynamic range of the assay for navoximod was 1-1000 ng/mL. The intra-run and inter-run precision was within 6.1% and accuracy was within ±9.6% of the nominal concentration values. Navoximod was found to be stable in human plasma over 5 freeze-thaw cycles, at least for 110 days at − 10 °C to − 30 °C and − 60 °C to − 80 °C, and for 24 h at room temperature. PK parameters were estimated using non-compartmental analysis (Phoenix WinNonlin 6.4; Certara, Princeton, NJ).

### Tumor response assessments

Objective tumor assessments by the investigators, including an assigned radiologist and confirmed by the clinical investigators, were conducted using CT or MRI studies obtained at screening, at Cycle 4, and approximately every 4th cycle thereafter, according to RECIST v1.1 and immune related response criteria (irRC). Endpoints included complete or partial response and stable disease, which were confirmed by tumor imaging.

### Plasma biomarker assessments

Blood was collected to monitor changes in plasma Kyn and Trp levels as peripheral markers of IDO1 activity modulation. Samples were collected at the same schedule as PK assessments, with another sample drawn at the 1 week scheduled clinic visit (Day 8). After collection, blood samples were centrifuged at 2000xg for 15 min in a refrigerated centrifuge at 2-8^o^ C to harvest the plasma and plasma samples were stored at -70^o^ C until analysis. Validated LC/MS-MS assays were used to measure the concentration of Kyn and Trp in plasma samples, with lower limits of quantitation of 25 ng/mL and 500 ng/mL, respectively. The intra-run and inter-run precision was within 4.7% and accuracy was within ±4.5% of the nominal concentration values for both analytes. Samples were analyzed at Covance Laboratories, Madison, WI.

### Statistical analyses

No formal hypotheses were tested in this study, and all analyses were descriptive and exploratory. Design considerations were not made with regard to explicit power and type I error, but to obtain preliminary safety, activity, pharmacokinetic, and pharmacodynamic information. For the safety and activity analyses, all patients who received ≥1 dose of navoximod were included. The number of patients enrolled at each dose level and the number of dose cohorts were determined by the safety profile observed during the course of the study.

## Results

The study was initiated in April 2014, enrolled 22 patients in 7 Cohorts (navoximod 50-800 mg BID on two treatment schedules) and was terminated early by the Sponsor in December 2016. The study was closed before completing planned enrollment as a result of increasing challenges to identifying suitable patients for the study.

### Patient characteristics

All the data presented herein are based on a data cut of Nov 16, 2016. A total of 22 patients were enrolled and received ≥1 dose of navoximod. The baseline characteristics of the patient population are shown in Table 1. The median age was 61 years with more males (68%) enrolled than females. The majority of patients were white (73%). Thirteen patients (59%) had an ECOG score of 1 at baseline. Colorectal cancer (27%) made up the highest proportion of malignancies, followed by head and neck cancer (14%). All patients had received ≥1 prior systemic therapy with a median of 3 (range 1-9), and 18 patients received ≥1 prior course of radiation therapy.

**Table 1:** Patient baseline and disease characteristics

Variable	Navoximod 21/28 days BID						Navoximod 28/28 days BID	All patients (*n* = 22)
	50 mg (*n* = 1)	100 mg (*n* = 3)	200 mg (*n* = 3)	400 mg (*n* = 3)	600 mg (*n* = 3)	800 mg (*n* = 6)	600 mg (*n* = 3)	
Age (yr), median (range)	65 (65-65)	58 (50-82)	63 (32-75)	54 (41-69)	58 (27-59)	69 (38-70)	67 (43-72)	61 (27-82)
Sex								
Female	1 (100%)	–	3 (100%)	3 (100%)	3 (100%)	3 (50%)	2 (67%)	15 (68%)
Male	–	3 (100%)	–	–	–	3 (50%)	1 (33%)	7 (32%)
ECOG performance status								
0	1 (100%)	1 (33%)	3 (100%)	–	1 (33%)	1 (17%)	2 (67%)	9 (41%)
1	–	2 (67%)	–	3 (100%)	2 (67%)	5 (83%)	1 (33%)	13 (59%)
Most common tumor types								
Colorectal	–	–	–	2 (67%)	–	4 (67%)	–	6 (27%)
Head and neck	–	–	1 (33%)	1 (33%)	–	1 (17%)	–	3 (14%)
Leiomyosarcoma	–	1 (33%)		–	–	–	1 (33%)	2 (9%)
Lung	–	1 (33%)	1 (33%)	–	–	–	–	2 (9%)
Pancreas	–	–	1 (33%)	–	1 (33%)	–	–	2 (9%)
Renal	–	–	–	–	1 (33%)	1 (17%)	–	2 (9%)
Bladder	1 (100%)	–	–	–	–	–	–	1 (5%)
Breast	–	–	–	–	–	–	1 (33%)	1 (5%)
Cholangiocarcinoma	–	1 (33%)	–	–	–	–	–	1 (5%)
Mesothelioma	–	–	–	–	–	–	1 (33%)	1 (5%)
Testicular	–	–	–	–	1 (33%)	–	–	1 (5%)
Number of prior systemic therapies, median (range)^a^	5 (5-5)	4 (1-5)	3 (1-3)	4 (3-4)	3 (2-9)	3.5 (2-6)		

*ECOG* Eastern Cooperative Oncology Group

^a^All patients underwent prior systemic therapy

### Dose escalation of navoximod

Across all dose cohorts of patients (50-800 mg BID), the median number of 28-day cycles of navoximod received per patient was 3 (range 1–20) and patients remained on study treatment a median of 75 days (range 2–552 days).

### Safety, tolerability, and adverse events

All patients experienced ≥1 AE during the study regardless of attribution to navoximod (Table 2). The MTD was not reached. A DLT was reported in 1 patient (69 year old male) in Cohort 6 (800 mg BID, 21/28 days schedule) with metastatic renal cell carcinoma. This patient experienced Grade 1 diarrhea with dark stool at Cycle 1, Day 14, Grade 3 hypotension that worsened to Grade 4, and Grade 4 lower gastrointestinal hemorrhage, treated with blood transfusion and vasopressors, and was assessed as possibly related to navoximod. However, even though the relationship to the study treatment could not be ruled out, this patient exhibited widespread peritoneal and GI serosal metastasis on baseline CT scan that could explain the symptoms.

**Table 2:** All Grade AEs in ≥10% of patients regardless of attribution to navoximod

	Navoximod 50 mg BID (*n* = 1)	Navoximod 100 mg BID (*n* = 3)	Navoximod 200 mg BID (*n* = 3)	Navoximod 400 mg BID (*n* = 3)	Navoximod 600 mg BID (*n* = 3)	Navoximod 800 mg BID (*n* = 6)	All Patients 21/28 day schedule (*N* = 19)	Navoximod continuous schedule 600 mg BID (*n* = 3)	All Patients (*N* = 22)
Any adverse event	1 (100%)	3 (100%)	3 (100%)	3 (100%)	3 (100%)	6 (100%)	19 (100%)	3 (100%)	22 (100%)
Fatigue	1 (100%)	2 (67%)	3 (100%)	1 (33%)	1 (33%)	3 (50%)	11 (58%)	2 (67%)	13 (59%)
Cough	1 (100%)	3 (100%)	–	–	1 (33%)	4 (67%)	9 (47%)	–	9 (40%)
Decreased appetite	1 (100%)	2 (67%)	3 (100%)	–	–	3 (50%)	9 (47%)	–	9 (40%)
Pruritus	–	2 (67%)	2 (67%)	1 (33%)	2 (67%)	1 (17%)	8 (42%)	1 (33%)	9 (40%)
Nausea	1 (100%)	3 (100%)	–	1 (33%)	1 (33%)	2 (67%)	8 (42%)	–	8 (36%)
Vomiting	–	1 (33%)	1 (33%)	1 (33%)	1 (33%)	1 (17%)	5 (26%)	1 (33%)	6 (27%)
Anxiety	1 (100%)	1 (33%)	1 (33%)	–	–	1 (17%)	4 (21%)	–	4 (18%)
AST increased	–	–	1 (33%)	1 (33%)	–	2 (33%)	4 (21%)	–	4 (18%)
Constipation	–	–	2 (67%)	–	–	2 (33%)	4 (21%)	–	4 (18%)
Dyspepsia	–	–	2 (67%)	2 (67%)	–	–	4 (21%)	–	4 (18%)
Dyspnea	1 (100%)	1 (33%)	–	–	–	2 (33%)	4 (21%)	–	4 (18%)
Wheezing	1 (100%)	1 (33%)	–	–	1 (33%)	1 (17%)	4 (21%)	–	4 (18%)
Rash terms ^a^	–	1 (33%)	–	1 (33%)	1 (33%)	1 (33%)	4 (21%)	1 (33%)	5 (23%)
Abdominal pain	–	2 (67%)	–	–	–	1 (17%)	3 (16%)	1 (33%)	4 (18%)
Hypokalemia	1 (100%)	–	–	–	1 (33%)	1 (17%)	3 (16%)	1 (33%)	4 (18%)
Ascites	–	–	–	1 (33%)	1 (33%)	1 (17%)	3 (16%)	–	3 (14%)
Neoplasm progression	–	–	1 (33%)	1 (33%)	1 (33%)	–	3 (16%)	–	3 (14%)
Upper respiratory tract infection	–	1 (33%)	1 (33%)	–	–	1 (17%)	3 (16%)	–	3 (14%)
Dry mouth	–	–	–	–	2 (67%)	–	2 (11%)	1 (33%)	3 (14%)

^a^Rash terms = rash and rash maculopapular

Grade ≥ 3 AEs regardless of attribution were reported in 14 (64%) patients (Table 3) and Grade ≥ 3 AE related to navoximod were reported in two patients (9%): Grade 4 lower gastrointestinal hemorrhage described above, and Grade 3 diverticulitis on Day 155 in a patient with leiomyosarcoma treated at 600 mg BID on the continuous dosing schedule.

**Table 3:** Grade ≥ 3 AEs regardless of attribution to navoximod

	Navoximod 50 mg BID (*n* = 1)	Navoximod 100 mg BID (*n* = 3)	Navoximod 200 mg BID (*n* = 3)	Navoximod 400 mg BID (*n* = 3)	Navoximod 600 mg BID (*n* = 3)	Navoximod 800 mg BID (*n* = 6)	All Patients 21/28 day schedule (*N* = 19)	Navoximod continuous schedule 600 mg BID (*n* = 3)	All Patients (*N* = 22)
Any adverse event	1 (100%)	3 (100%)	1 (33%)	3 (100%)	2 (67%)	2 (33%)	12 (63%)	2 (67%)	14 (63%)
Neoplasm progression	–	–	1 (33%)	1 (33%)	1 (33%)	–	3 (16%)	–	3 (14%)
AST increased	–	–	1 (33%)	1 (33%)		–	2 (11%)	–	2 (9%)
Anemia	–	–	–	–	1 (33%)	–	1 (5%)	–	1 (5%)
Ascites	–	–	–	–	1 (33%)	–	1 (5%)	–	1 (5%)
Dysphagia	1 (33%)	–	–	–		–	1 (5%)	–	1 (5%)
Hypokalemia	–	–	–	–		1 (17%)	1 (5%)	–	1 (5%)
Hypotension	–	–	–	–		1 (17%)	1 (5%)	–	1 (5%)
Lower GI hemorrhage	–	–	–	–		1 (17%)	1 (5%)	–	1 (5%)
Mental status changes	–	–	–	1 (33%)		–	1 (5%)	–	1 (5%)
Muscular weakness	–	–	–	–	1 (33%)	–	1 (5%)	–	1 (5%)
Peripheral neuropathy	–	–	–	1 (33%)	–	–	1 (5%)	–	1 (5%)
Acute pancreatitis	–	1 (33%)	–	–	–	–	1 (5%)	–	1 (5%)
Pneumonia	–	1 (33%)	–	–	–	–	1 (5%)	–	1 (5%)
Small Intestinal Obstruction	–	1 (33%)	–	–	–	–	1 (5%)	–	1 (5%)
Tumor pain	–	–	–	–	1 (33%)	–	1 (5%)	–	1 (5%)
Bloody discharge	–	–	–	–	–	–	–	1 (33%)	1 (5%)
Diverticulitis	–	–	–	–	–	–	–	1 (33%)	1 (5%)
Lymphopenia	–	–	–	–	–	–	–	1 (33%)	1 (5%)

Review of electrocardiograph data collected at baseline, Cycle 1, Day 1, and Cycle 1, Day 8 did not suggest a risk of QT prolongation with navoximod. Liver function test abnormalities (increased aspartate aminotransferase and alanine aminotransferase) were observed in a total of four patients (18%).

Two patients required dose modifications: 1 dose interruption in the 100 mg BID cohort (Grade 1 tachycardia, dyspnea, and nausea), and another dose interruption/reduction from 800 to 600 mg BID due to Grade 2 AST/ALT and Grade 2 maculopapular rash. No AEs requiring withdrawal of study drug were reported. Reasons for discontinuation from study treatment included disease progression (20 patients), physician’s decision (1 patient) and patient withdrawal (1 patient). Three patients died during the 30-day safety follow-up due to disease progression.

### Pharmacokinetic analysis

**Fig. 2:**
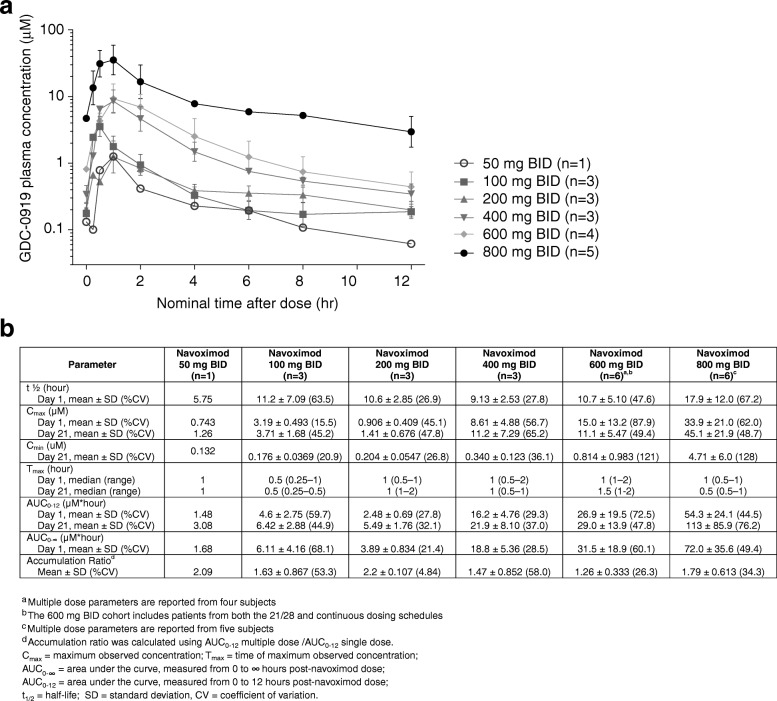
**a** Mean (± SD) plasma concentrations of navoximod after multiple twice-daily doses on Cycle 1, Day 21. The 600 mg BID cohort includes patients from both the 21/28 and continuous dosing schedules. **b** Single dose and multiple dose pharmacokinetic parameters for navoximod

Under fasting conditions, navoximod was rapidly absorbed (T_max_ ~ 1 h) and demonstrated linear and dose proportional increases in exposure, with an average half-life across all dose levels of ~ 12 h, which is supportive of BID dosing (Fig. 2). The mean accumulation index (MD AUC_0-12_/SD AUC_0-12_) was ~ 1.7, consistent with its half-life and a BID dosing interval.

### Clinical activity

**Fig. 3:**
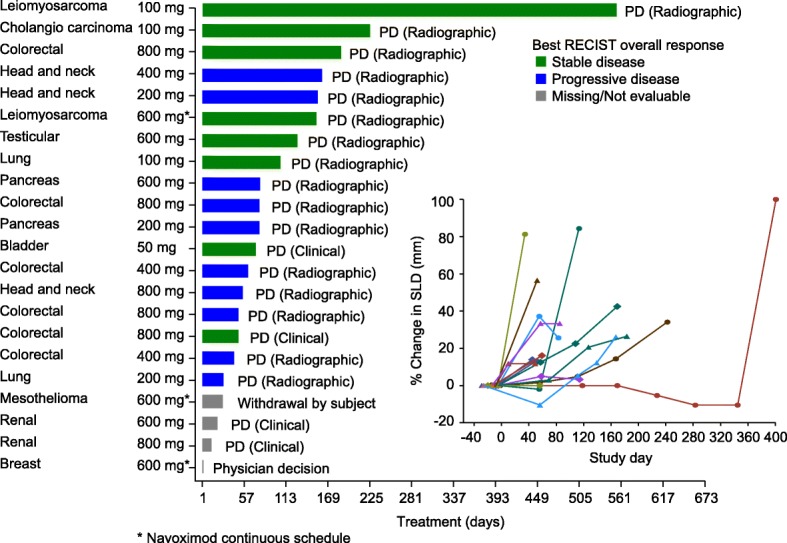
Time on study treatment (navoximod dose BID), reason for treatment discontinuation, and best response by RECIST/change in sum of longest diameters for individual patients. PD: Progressive disease

Time on study treatment, reasons for treatment discontinuation, and best response are shown in Fig. 3. No post-baseline tumor assessments were available for four patients who discontinued due to clinical progression prior to the initial tumor assessment. Based on RECIST v1.1 there were no objective responses, and the best response was limited to stable disease (SD) in 8/22 (36%) patients who were distributed amongst all dose cohorts. Ten patients (45%) had progressive disease.

Based on irRC there were no objective responses, and the rate of SD was 12/22 (68%) based on tumor assessments in target lesions only (irRC requires confirmation of PD 4 weeks after initial assessment). One patient only had non-target lesions at baseline and could not be assessed via irRC.

One patient with a diagnosis of leiomyosarcoma of the colon remained on study treatment for over 13 months with best response of SD. This patient was enrolled with presumed tumor progression after prior chemotherapy, but upon re-examination of baseline CT scans tumor progression prior to study entry was not confirmed.

The lack of objective responses is consistent with preclinical data which indicates that IDO inhibition is only effective in the context of chemotherapy, vaccination, radiation and/or checkpoint inhibition. Moreover, these results parallel those observed for epacadostat as single agent, which reported no objective responses and 35% SD response by RECIST [17].

### Pharmacodynamics

**Fig. 4:**
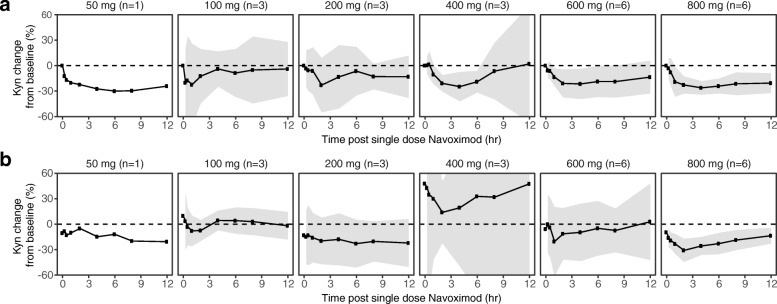
Mean (± 95% CI) fold changes in plasma Kyn at (**a**) Cycle 1, Day 1 and (**b**) Cycle 1, Day 21, relative to Day 1 predose levels after a single oral dose of navoximod. Gray ribbons represent 95% confidence intervals, and dashed horizontal lines represent no change from baseline. The significance of the Kyn changes from baseline is indicated by the 95% confidence intervals relative to the horizontal lines representing no change. If the confidence interval does not overlap with the no change reference line, then the change is significant in the context of a 95% confidence interval

At higher doses including 400, 600, and 800 mg BID, navoximod decreased plasma Kyn from baseline levels by 25-30%, 2-4 h after dosing, with kinetics that were consistent with the half-life of the drug (Fig. 4a). In the context of 95% confidence intervals (colored in gray), the mean change from baseline was statistically significant at the 2, 4, and 6 h post-dose timepoints in the 400 mg BID cohort, at the 1, 2, and 4 h post-dose timepoints in the 600 mg BID cohort, and at all timepoints including 1 h post-dose and beyond in the 800 mg BID cohort. At 800 mg BID, similar maximal decreases in Kyn were observed after multiple doses of navoximod (Fig. 4b). No significant modulation of plasma Trp levels was observed on days 1 or 21 (results not shown).

Navoximod has an in vitro potency against the recombinant purified human IDO enzyme of 28 nM (IC50) with a K_*i*_ of 5.8 nM. Its potency in cellular assays expressing human IDO is EC_50_ of 70 nM and an EC_75_ of ~ 200 nM and it restores IDO-mediated suppression of CD8^+^ T cell proliferation in a human MLR assay with an EC_50_ of 90 nM and an EC_90_ of ~ 200 nM [22]. Considering that navoximod has a %PPB of ~ 50% (fu = 0.5) it could be expected that concentrations that are > 400 nM should achieve > 75% of IDO-mediated Kyn synthesis inhibition and > 90% restoration of T cell proliferation in vivo.

The exposures achieved in this study would thus be expected to maintain trough free drug concentrations above the cellular EC_50_, at doses of 600 mg or higher (Fig. 2). This is consistent with the observation of more pronounced Kyn suppression in the higher dose cohorts of this study, which indicates that plasma exposures obtained at doses higher than 600 mg may be necessary for optimal systemic modulation of Kyn production in human subjects and for stimulation of T cell proliferation in vivo.

## Discussion

Navoximod was well-tolerated at doses up to 800 mg BID on a 21/28 day schedule or 600 mg on continuous dosing, with the MTD not reached. The study was closed before completing planned enrollment as a result of increasing challenges to identifying suitable patients for the study. Navoximod was rapidly absorbed (T_max_ ~ 1 h) and had dose proportional increases in exposure, with a half-life supportive of BID dosing. Best response based on RECIST v1.1 was limited to SD in 8 out of 18 patients with post-baseline tumor assessments. At doses higher than 400 mg BID, plasma Kyn was modulated in a manner consistent with the half-life of the drug and predicted activity based on considerations of potency in in vitro cellular assays and drug free fraction in plasma, with maximal Kyn decreases observed at post-dose timepoints following the T_max_ by a few hours, indicating target engagement with peripheral PD effects.

Initially the study completed enrollment of patients at the 21/28 day schedule up to maximum administered dose (MAD) of 800 mg BID. Doses as low as 100 mg BID achieved a trough concentration at 12 h above the cellular EC_50_ of 90 nM, while doses of 800 mg BID achieved a trough concentration of 4.71 μM. A 21/28 schedule was initially selected to prevent desensitization to IDO inhibitors after continued IDO inhibition, as is seen in IDO-KO mice [27–29], and to mitigate the potential for autoinduction of metabolism observed in rats and dogs (Genentech, data on file). PK data from the 21/28 schedule demonstrated an accumulation ratio consistent with the dosing frequency, suggesting that navoximod does not exhibit autoinduction in humans. Therefore, the study was amended to include the evaluation of safety, PK, and peripheral and tumor PD at a 28/28 day schedule at 600 mg BID. Upon limited enrollment of three patients over the course of 1 year the sponsor decided to close the study for limited feasibility of identifying suitable patients for single agent navoximod. Continued safety and PD evaluations were deferred to a parallel Phase Ib study combining continuous navoximod dosing with the anti-PD-L1 agent atezolizumab.

Immune-related AEs have been reported for almost all organ systems after the use of marketed checkpoint inhibitors, including liver toxicity and dermatologic reactions [30]. In this study, none of the AEs were reported as immune-related; however, 1 patient experienced both liver enzyme elevations and rash. A possible relationship between study treatment and elevation of liver enzymes cannot be ruled out and will continue to be monitored in additional studies with navoximod. Rash events were reported in five patients (23%) overall, which included the terms of rash (three patients, 14%) and maculo-papular rash (two patients, 9%). All events were Grade 1-2.

Currently available immune modulators can be effective given as single agents, albeit in a small percentage of patients. The accumulated data targeting the IDO pathway suggests that IDO inhibitors produce minimal antitumor activity when given alone as evidenced by the current study and the Phase I trials of epacadostat and indoximod [17, 31]. Checkpoint inhibitors of PD-1/PD-L1 show activity as a single agent, though with response rates of less than 20%, cures occurring in less than 5% of patients in most indications and somewhat increased responsiveness in select indications, such as melanoma, microsatellite instability-high tumors, and Hodgkin’s disease [9]. Therefore, combination therapy regimens employing one or more targeted immunotherapy drugs may be required to fully activate the host immune system.

Emerging clinical data from Phase I/II studies suggest the potential for increased benefit from a combination approach with IDO inhibitors. Epacadostat, given in combination with ipilimumab showed the potential for enhanced melanoma patient outcomes compared to ipilimumab monotherapy [32], and epacadostat or indoximod given with pembrolizumab showed promising results in melanoma and solid tumors [18–21, 33]. The preliminary results from the Phase I/II study of daily epacadostat and pembrolizumab in treatment naïve melanoma patients showed an objective response rate of 56% (15% complete) [34]. These results have led to the initiation of a Phase III trial of the combination in patients with melanoma. A 61% response rate was observed in 51 patients with cutaneous melanoma in an early study of indoximod in combination with pembrolizumab [33]. The complete remission rate was 20% in this study. In a small randomized study of indoximod after sipuleucel vaccine therapy, progression free survival was improved by 6.2 months [35]. The median radiographic progression free survival was 10.3 months compared with 4.1 months in the placebo arm. However, there was no increase in T cell response to PA2024 by ELISPOT or ELISA assay. Additionally, there were no changes in PSA between groups.

In conclusion, navoximod is a novel checkpoint inhibitor with potential immune modulating properties. In this trial we observed liver enzymes elevations and cutaneous toxicities (rash) that should be monitored in further studies that, given evidence of peripheral PD effects via suppression of Kyn as a single agent, warrant further investigation of navoximod in combination with other therapies. A Phase Ib study of navoximod in combination with atezolizumab in patients with solid tumors is currently ongoing (NCT02471846). Preliminary data from dose escalation stage of that study reported a response rate of 9% in an unselected patient population [36].

## Abbreviations

AE: Adverse event
AUC: Area under the curve
BID: Twice daily
C_max_: Maximum observed concentration
CV: Coefficient of variation
DLT: Dose-limiting Toxicity
ECOG: Eastern Cooperative Oncology group
IDO1: Indoleamine-2,3-dioxygenase 1
IHC: Immunohistochemistry
irRC: Immune-related response criteria
Kyn: Kynurenine
LC/MS-MS: Liquid chromatographic-tandem mass spectrometry
MAD: Maximum administered dose
MTD: Maximum tolerated dose
PD: Pharmacodynamics
PD: Progressive disease
PD-1: Programmed cell death protein 1
PD-L1: Programmed death-ligand 1
PK: Pharmacokinetics
SD: Stable disease
t_1/2_: Half-life
T_max_: Time of maximum observed concentration
Trp: L-tryptophan
